# Supplementary figures and images for: The chromatin remodeling protein CHD-1 and the EFL-1/DPL-1 transcription factor cooperatively down regulate CDK-2 to control SAS-6 levels and centriole number
Source: PLoS Genet. 2022 Apr 4;18(4):e1009799. doi: 10.1371/journal.pgen.1009799 (PMC9009770; doi:10.1371/journal.pgen.1009799)

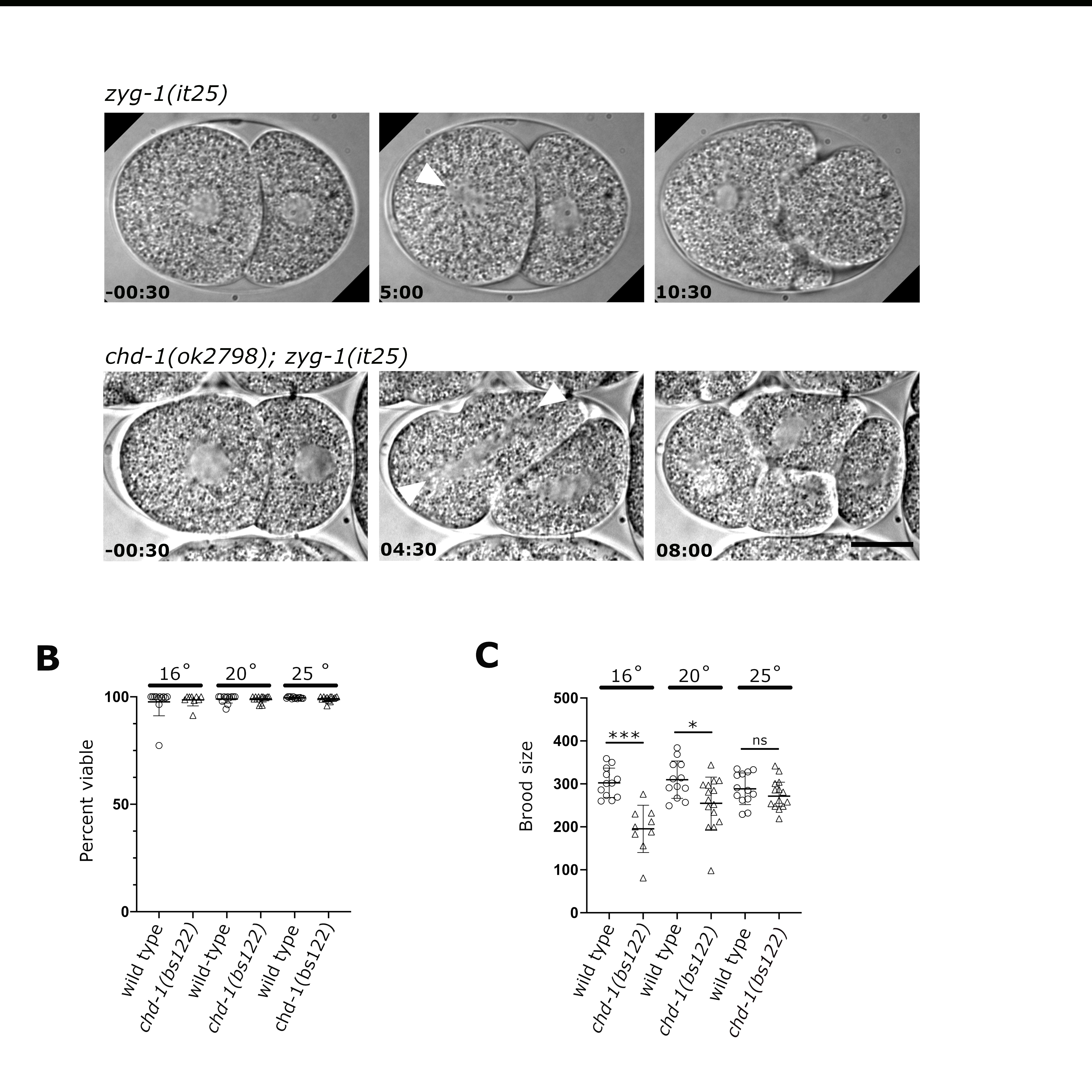

Supplement: S1 Fig — (A) Frames from 4D DIC imaging of a zyg-1(it25) embryo (top) and a chd-1(ok2798); zyg-1(it25) double mutant embryo (bottom). The first time point shows each embryo early at the two-cell stage. The second time point shows embryos in second mitosis with spindle poles in AB cell indicated by arrowheads. The third time point shows embryos after exiting second mitosis. The zyg-1(it25) embryo assembles monopolar spindles and fails to exit the two-cell stage while the chd-1(ok2798); zyg-1(it25) embryo assembles bipolar spindles and progress to the four-cell stage. Time is in min:sec and is relative to AB nuclear envelope breakdown (t = 0). Scale bar, 10 μm. (B) Embryonic viability among the offspring of chd-1(bs122) hermaphrodites measured at the indicated temperatures. Each data point represents the percent embryonic viability among the progeny of an individual hermaphrodite over the course of its reproductive lifespan. Bars indicate mean and standard deviation. (C) The brood sizes of chd-1(bs122) hermaphrodites were measured at the indicated temperature. Each dot represents the brood size of a single hermaphrodite. Bars indicate mean and standard deviation. *** p<0.0001, *p,0.05, ns = not significant, two-tailed t test. (TIF) [file pgen.1009799.s001.tif]

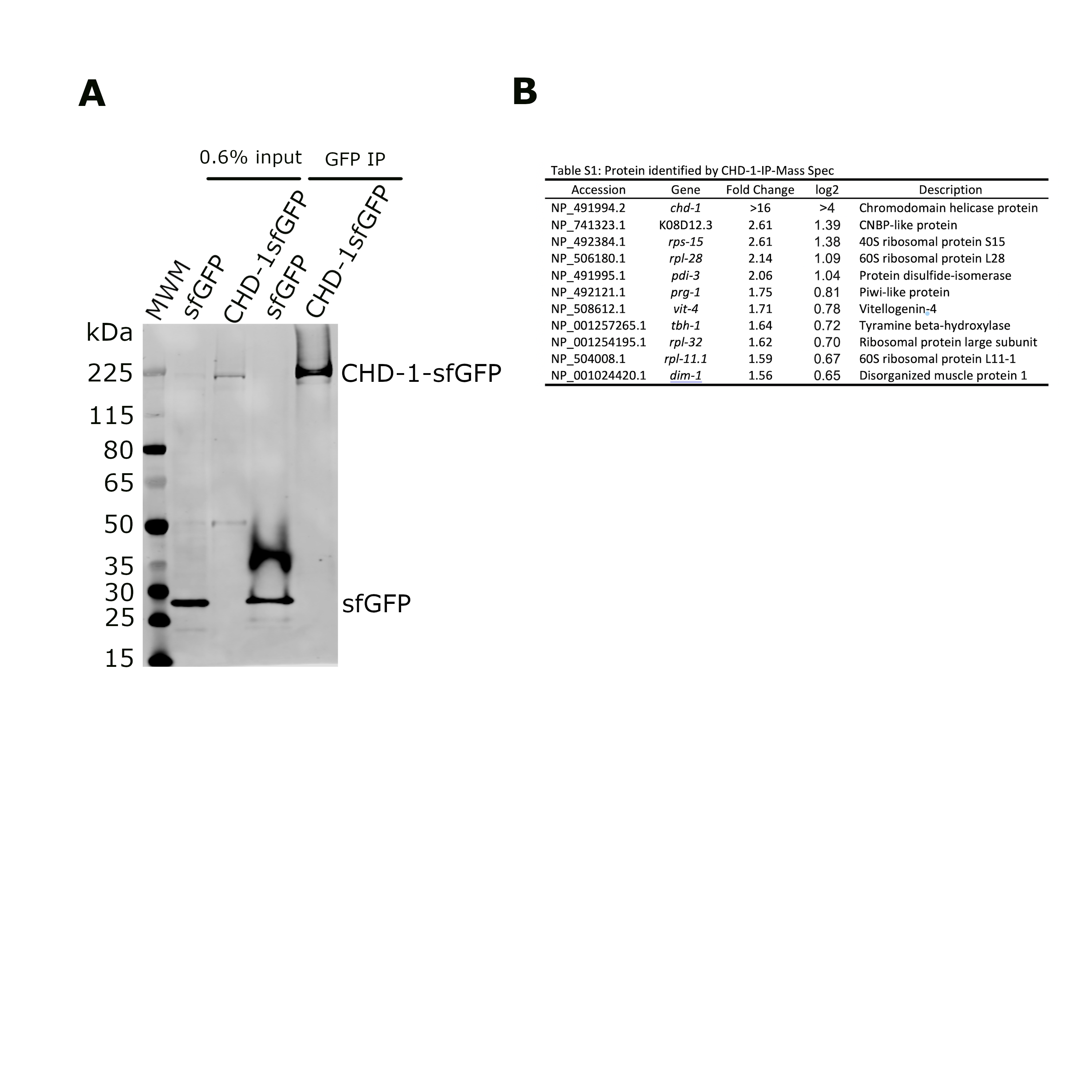

Supplement: S2 Fig — Strain OC829 (chd-1(bs122)) expresses sfGFP from the endogenous chd-1 locus and served as a negative control. Strain OC798 (chd-1(bs125)) expresses CHD-1::sfGFP from the endogenous locus. The blot was probed for GFP to validate the pull down. (B) Proteins identified as significantly enriched in the CHD-1::sfGFP immunoprecipitate. (TIF) [file pgen.1009799.s002.tif]

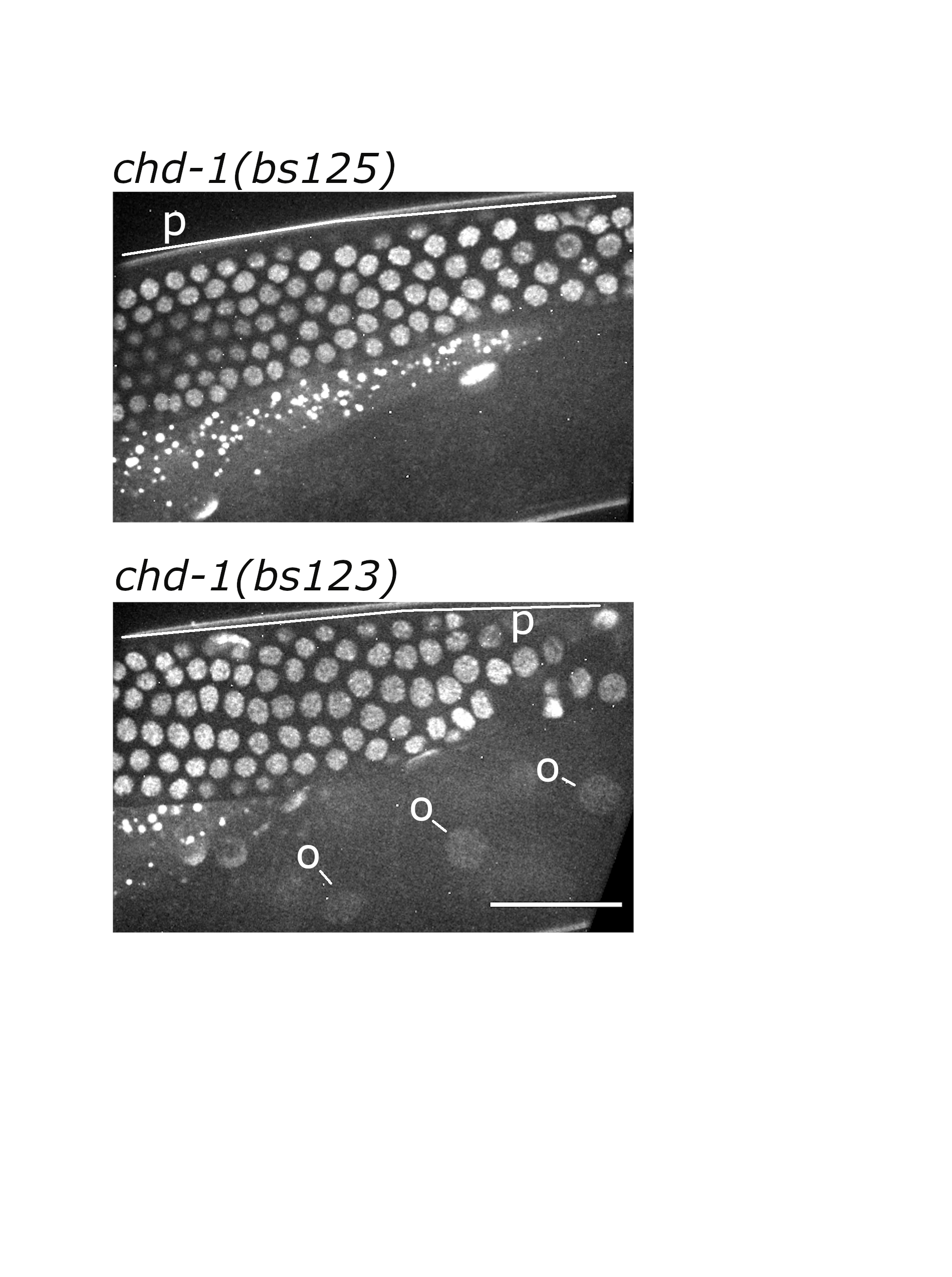

Supplement: S3 Fig — Both proteins localize similarly. Shown are nuclei of the distal germ line (p) and for CHD-1(D538N)::GFP, oocyte nuclei (o). The wild-type transgene is also expressed in oocyte nuclei but these nuclei are not present in the optical plane shown. Scale bar = 25μm. (TIF) [file pgen.1009799.s003.tif]

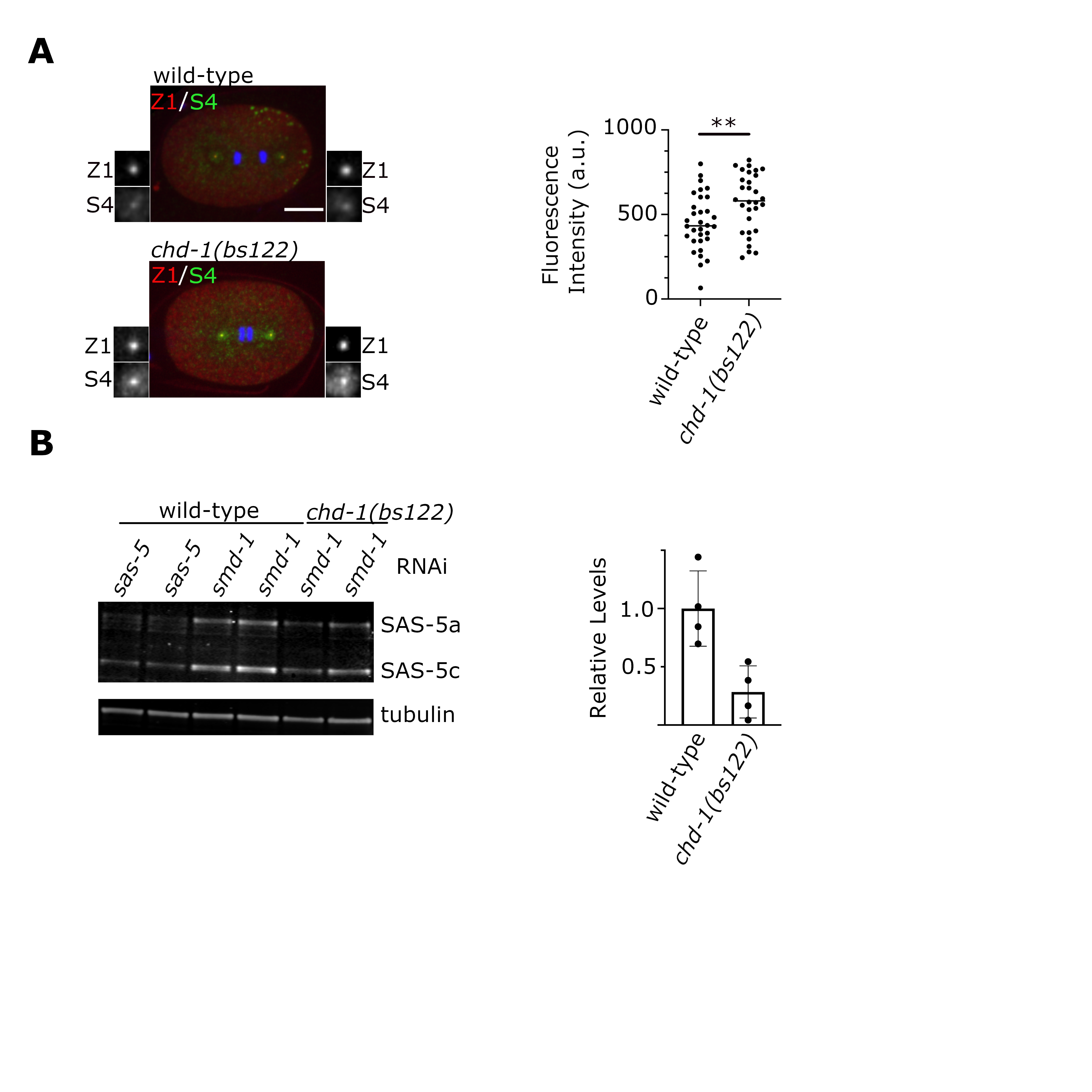

Supplement: S4 Fig — (A) Representative images of embryos immunostained for ZYG-1::SPOT (red), SAS-4 (green) and DNA (blue). Each image is a maximum intensity projection of 10 focal planes. Top left, a zyg-1(bs197[zyg-1::spot]) embryo and bottom left, a chd-1(bs122); zyg-1(bs197[zyg-1::spot]) embryo. Insets are three-fold magnified images of centrioles stained with ZYG-1(Z1) or SAS-4 (S4). Quantification of the raw integrated intensity (expressed in arbitrary units or a.u.) of ZYG-1::SPOT staining at centrioles. Each dot represents a single centriole in anaphase. Bar = 10 μm. **p = 0.007, unpaired t test (B). Left, quantitative immunoblot of SAS-5 showing isoforms a and b. Each lane is an independent sample of 100 gravid adults. Right, Quantitation of SAS-5a showing relative levels in wild-type and chd-1(bs122) animals. (TIF) [file pgen.1009799.s004.tif]

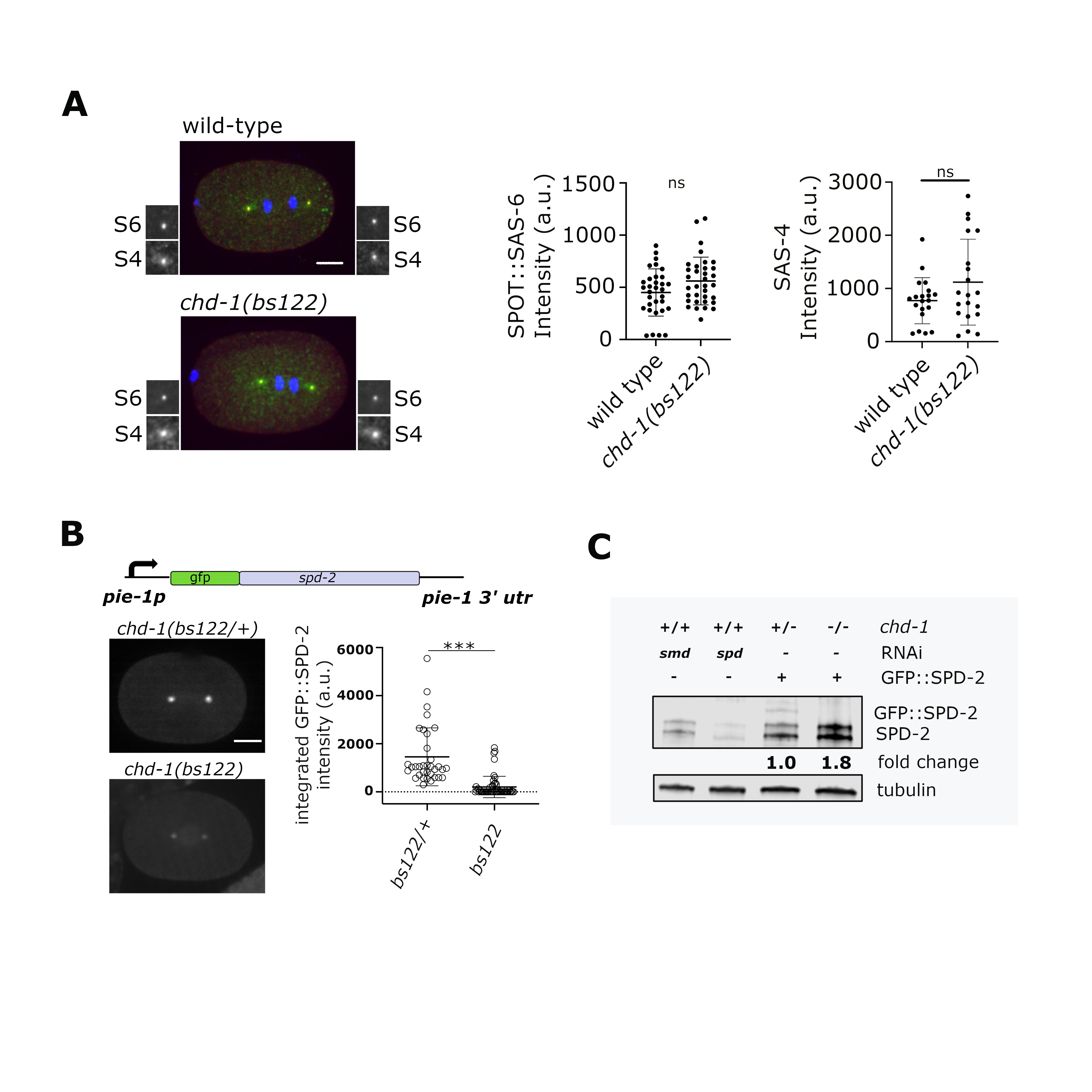

Supplement: S5 Fig — (A) Left. Representative images of embryos immunostained for SPOT::SAS-6 (red), SAS-4 (green) and DNA (blue). Each image is a maximum intensity projection of 25 focal planes. Top, a sas-6(bs188 [spot::SAS-6]) embryo and bottom, a chd-1(bs122); sas-6(bs188 [spot::SAS-6]) embryo. Insets are 1.5-fold magnified images of centrioles stained for SPOT::SAS-6 (S6) or SAS-4 (S4). Scale bar, 10 μm. Right. Quantification of the raw integrated intensity (expressed in arbitrary units or a.u.) of SPOT::SAS-6 and SAS-4 at centrioles. Each dot represents a single centriole in anaphase. Not significant (ns) as determined by an unpaired t test. (B) Quantitative fluorescence microscopy of a gfp::spd-2 transgene in chd-1(bs122) heterozygous (control) and homozygous embryos. As shown in the schematic, the transgene is expressed under control of the pie-1 promoter and 3’ utr and its expression is negatively affected by loss of CHD-1. Representative images (left, scale bar = 10 μm) and quantitation (right) are shown. Each dot represents a single centrosome. Bars indicate mean and standard deviation. ***p<0.0001, unpaired t test with Welch’s correction. (C) Quantitative immunoblot of endogenous SPD-2 and GFP::SPD-2 levels in chd-1(bs122) heterozygotes and homozygotes. Note that endogenous SPD-2 is elevated almost two-fold in the chd-1(bs122) homozygotes relative to heterozygous siblings, while the level of GFP::SPD-2 is reduced. (TIF) [file pgen.1009799.s005.tif]

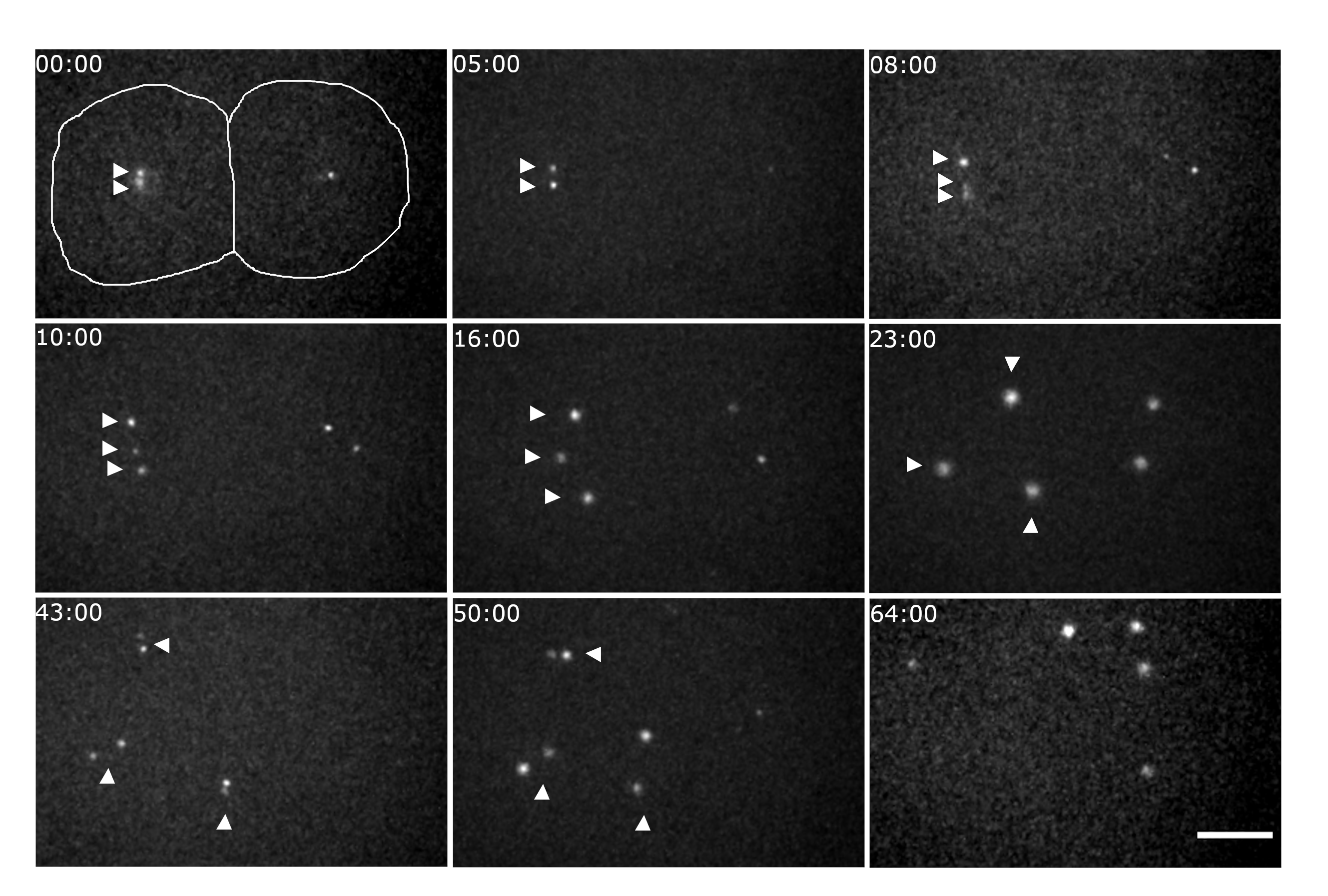

Supplement: S6 Fig — Select time points from a recording of a chd-1(bs122); dpl-1(bs169) embryo expressing a spd-2::mCherry transgene that was grown and recorded at 12.5° C. The first time point is at the onset of the two-cell stage. Initially, two SPD-2::mCherry-positive bodies are visible in the anterior blastomere (arrowheads, 00:00 and 05:00). Shortly thereafter, a third SPD-2::mCherry-positive body becomes visible (arrowheads, 08:00 and 16:00). These structures mature into centrosomes that form a tripolar spindle (23:00). Subsequently, each centrosome resolves into two structures (43:00 and 50:00), indicating that all three centrosomes are capable of duplication. The two centrosomes of the posterior blastomere are visible from timepoints 08:00 to 23:00. Each image is a maximum intensity projection of 6 focal planes. Time is in minutes: seconds and is relative to first frame. Scale bar = 10 μm. Right. (TIF) [file pgen.1009799.s006.tif]
