# Supplementary material for: The chromatin remodeling protein CHD-1 and the EFL-1/DPL-1 transcription factor cooperatively down regulate CDK-2 to control SAS-6 levels and centriole number
Source: PLoS Genet. 2022 Apr 4;18(4):e1009799. doi: 10.1371/journal.pgen.1009799 (PMC9009770; doi:10.1371/journal.pgen.1009799)
Supplement: S3 Table — (DOCX) [file pgen.1009799.s009.docx]

| **Table S3: *C. elegans* strains** | | | |
| --- | --- | --- | --- |
| **Strain name** | | | **Genotype** |
| N2 | | wild type | |
| OC661 | | *chd-1(ok2798) I* (backcrossed 6x) | |
| OC829 | | *chd-1(bs122) I* | |
| OC14 | | *zyg-1(it25) II* | |
| OC667 | | *chd-1(ok2798) I; zyg-1(it25) II* (line 1) | |
| OC668 | | *chd-1(ok2798) I; zyg-1(it25) II* (line 2) | |
| OC818 | | *chd-1(bs122) I; zyg-1(it25) II* | |
| OC869 | | *bsSi15* [*pKO109: spd-2p-spd-2::mCherry::spd-2 3'-utr, unc-119(+)*] *I; zyg-1(it25) bsSi30*[*pCW9: unc-119(+) pcdk-11.2::sfgfp::his-58::cdk-11.2 3' utr*] *II* | |
| OC971 | | *bsSi15* [*pKO109: spd-2p-spd-2::mCherry::spd-2 3'-utr, unc-119(+)*] *chd-1(bs185) I; zyg-1(it25) bsSi30[pCW9: unc-119(+) pcdk-11.2::sfgfp::his-58::cdk-11.2 3' utr] II* | |
| OC798 | | *chd-1(bs125* [*chd-1::sfgfp*]*) I* | |
| OC819 | | *chd-1(bs125*[*chd-1::sfgfp*]*) I; zyg-1(it25) II* | |
| OC821 | | *chd-1(bs123*[*chd-1::sfgfpD538N*]*) I* | |
| OC922 | | *chd-1(bs123*[*chd-1(D538N)::sfgfp*]*) I; zyg-1(it25) II* | |
| OC993 | | *chd-1(bs122)/ hT2*[*bli-4(e937) let-?(q782) qIs48*]  *(I;III); bsIs2* [*pCK5.5: Ppie-1::gfp::spd-2*] | |
| OC947 | | *dpl-1(bs169) II* | |
| OC490 | | *zyg-1(it25) dpl-1(bs21) II* | |
| OC965 | | *chd-1(bs122); dpl-1(bs169)/mnC1*[*mIs14 dpy-10(e128)*] *II* | |
| OC1002 | | *zyg-1 (bs197 [zyg-1::spot]) II* | |
| OC996 | | *chd-1(bs122) I; zyg-1 (bs197 [zyg-1::spot]) II* | |
| OC973 | | *sas-6(bs188 [spot::SAS-6]), fem-1 (hc17 ts)IV, dpy-20 (e1282)IV* | |
| OC989 | | *chd-1(bs122) I; sas-6(bs188 [spot::SAS-6])IV, fem-1 (hc17 ts)IV, dpy-20 (e1282)IV* | |
| OC1057 | | *cdk-2(kim31[cdk-2::aid::3xflag]) I; eSi38[sun-1p::TIR1::mRuby::sun-1 3'UTR + Cbr-unc-119(+)] IV* | |
| OC779 | | *bsSi15 [pKO109: spd-2p-spd-2::mCherry::spd-2 3'-utr, unc-119(+)] I; bsSi30[pCW9: unc-119(+) pcdk-11.2::sfgfp::his-58::cdk-11.2 3' utr] II; unc-119(ed3) III* | |
| OC1105 | | *bsSi15 [pKO109: spd-2p-spd-2::mCherry::spd-2 3'-utr, unc-119(+)] chd-1(bs185) I; bsSi30[pCW9: unc-119(+) pcdk-11.2::sfgfp::his-58::cdk-11.2 3' utr] II; unc-119(ed3) III* | |
| OC1107 | | *zyg-1(it25)*II; *fem-1(hc17ts) sas-6(bs188(spot::sas-6))*IV | |
| OC1108 | | *chd-1(bs122)*I; *zyg-1(it25)*II; *fem-1(hc17ts) sas-6(bs188(spot::sas-6))*IV | |
| OC1111 | | *bsSi15 [pKO109: spd-2p-spd-2::mCherry::spd-2 3'-utr, unc-119(+)] chd-1(bs185) I; dpl-1(bs169)/ )/mnC1*[*mIs14 dpy-10(e128)*] *II* | |
|  |  | | |
