# Supplementary material for: The chromatin remodeling protein CHD-1 and the EFL-1/DPL-1 transcription factor cooperatively down regulate CDK-2 to control SAS-6 levels and centriole number
Source: PLoS Genet. 2022 Apr 4;18(4):e1009799. doi: 10.1371/journal.pgen.1009799 (PMC9009770; doi:10.1371/journal.pgen.1009799)
Supplement: S4 Table — (DOCX) [file pgen.1009799.s010.docx]

| Table S4: CRISPR reagents | | |
| --- | --- | --- |
| Allele | Reagent | Sequence |
| *chd-1(bs122)* | 5’-crRNA | 5’-AUCAGUCUGAAUCAUCAUCU-3’ |
|  | 3’-crRNA | 5’-agaUggagUUagaCUAAUGU-3’ |
|  | Forward repair template | 5’-gggttttttgttgagaaaaaataagaatttca  gatATGGTGAGTAAAGGAGAAGAACTTTT-3’ |
|  | Reverse repair template | 5’-tgtgtgcaggtagacgaaaagatggagt  tagaCTActacttgtcatcgtcatccttgt-3’ |
| *chd-1(bs123)* | crRNA | 5’-AAGAUUCGUCGUUUUUGAGA-3’ |
|  | Repair template | 5’-CTGTGTGAGAGATTTGTAAAGCAAAGATT  CGTCGTTTTTGAGACGATGAGCTTCGTTAACAAG  AAGAGCTGCCCAATCAATTGATGACAGGAATGCT  TTATC-3’ |
| *chd-1(bs125)* | 5’-crRNA | 5’-TGGAGTTAGACTAATGTCGG -3’ |
|  | 3’-crRNA | 5’- AGATGGAGTTAGACTAATGT -3’ |
|  | Forward repair template | 5’-gtgtagtgtgtgcaggtagacgaaaagatggagttagaCTAC  TTGTAGAGCTCGTCCATTCCGTG-3’ |
|  | Reverse repair template | 5’-caacaacatttttttgatgtttaatattttcagGAGAAAA  ATCGTCGACATatgTCCAAGGGAGAGGAGCTCTTCACC-3’ |
| *chd-1(bs185)* | 5’-crRNA | 5’-AUCAGUCUGAAUCAUCAUCU-3’ |
|  | 3’-crRNA | 5’-agaUggagUUagaCUAAUGU-3’ |
|  | Repair template | 5’-TGTGTGCAGGTAGACGAAAAGATGGAGTTAGACTAATG  TCGAGATGATGATTCAGACTGATTCCACATATCTGAAATTC-3’ |
| *dpl-1(bs169)* | crRNA | 5’-GGUGGUCCACACCCGCCGGG-3’ |
|  | Repair template | 5’-GACATCAGTTACAACCTGGACAACGAATCGTAACTTAGC  GAATTGTTGCTCCATGGTGGTCCACACCCGCCGGGCACAATTGTTCGGAA-3’ |
| *zyg-1(bs197)* | crRNA | 5’-AGCAUUUCUGAAGCUCUGUC-3’ |
|  | crRNA | 5’-gUaggagcacaUgaagUaag-3’ |
|  | Repair template | 5’-ATCAACGATTTATGCGAACTGATCGAGTACCTGATAGAG  CCTCTGAAATGCTTCATACGCTGTGCGAACGTATGAGAAAACTACATCAACCAGACCGTGTCCGTGCCGTCTCCCACTGGTCCTCCTAATAGTAATAACTTAGTATACGATGAGTTTTCGTCTTACTTCATGTGCTCCTACATTTTACCACATATCTTT-3’ |
| *sas-6(bs188)* | crRNA | 5’-AUUUUGCUAGUCAUUUUUGU-3’ |
|  | Repair template | 5’-ATGAGTGTTTGATCGAATAATGCAATTTTGCTAGTGGAG  GACCAGTGGGAGACGGCACGGACACGGTCTGGCATTTTTGTGGGAGAAAATCTGAAAAAAATTATGT-3’ |
