# Supplementary material for: The chromatin remodeling protein CHD-1 and the EFL-1/DPL-1 transcription factor cooperatively down regulate CDK-2 to control SAS-6 levels and centriole number
Source: PLoS Genet. 2022 Apr 4;18(4):e1009799. doi: 10.1371/journal.pgen.1009799 (PMC9009770; doi:10.1371/journal.pgen.1009799)
Supplement: S5 Table — (DOCX) [file pgen.1009799.s011.docx]

| Table S5: qRT-PCR primer sequences | |
| --- | --- |
| Primer | Sequence |
| *act-1* Fwd | 5’-CAGAAGGAAATCACCGCTCTT-3’ |
| *act-1* Rev | 5’-ATAGATCCTCCGATCCAGACG-3’ |
| *cdk-2* Fwd | 5’-CCTGACTGGAAATGGTCTTGA-3’ |
| *cdk-2* Rev | 5’-GAAAGTAGCGATGAGAGAGAGC-3’ |
| *cki-2* Fwd | 5’-ATCTCCTCCTGTCGTGGAA-3’ |
| *cki-2* Rev | 5’-TCCTGTTGTTCGATTGATTGTTTAG-3’ |
| *sas-6* Fwd | 5’-GGAGGTTGATGCACGGAAA-3’ |
| *sas-6* Rev | 5’-GGAGTCTTCGAGAATAGCTCAAAT-3’ |
| *spd-2* Fwd | 5’-GCAGCATTAGAAGAAGCTCGTA-3’ |
| *spd-2* Rev | 5’-TCCAGAATGTCGTGCAGATG-3’ |
