# Supplementary material for: The chromatin remodeling protein CHD-1 and the EFL-1/DPL-1 transcription factor cooperatively down regulate CDK-2 to control SAS-6 levels and centriole number
Source: PLoS Genet. 2022 Apr 4;18(4):e1009799. doi: 10.1371/journal.pgen.1009799 (PMC9009770; doi:10.1371/journal.pgen.1009799)
Supplement: Attachment — Submitted filename: Response to reviewers comments.docx [file pgen.1009799.s013.docx]

List of major changes to paper and figures

1. Figure 1E redrew graph to show percentages of centrioles that duplicated.
2. Figure 1F Added graphics showing expected behavior of centrioles when duplication proceeds normally and when duplication is blocked. Added time stamps and color key.
3. Figure 1 legend was amended to account for changes to figure.
4. Created new Figure S2 by including image of immunoblot of Mass IP experiment and list of proteins identified from Table S1. Table S1 deleted. Renamed tables and figures accordingly and updated text citations.
5. Added percentages of multipolar spindles in Table 1
6. Added centrosome-associated SAS-4 quantitation to Figure S5 (old Figure S4). Adjusted text and figure legend accordingly.
7. Added DIC images from time-lapse recording of *zyg-1(it25)* and *chd-1(ok2798); zyg-1(it25)* embryos to document suppression of spindle assembly defect.
8. Added experiment showing that loss of CHD-1 results in an increase of centrosome associated SAS-6 in a *zyg-1(it25)* background to Figure 3 (panel D). Relocated original panels 3D and 3E to Figure S5 (new panels B and C). Adjusted text and figure legends accordingly.
9. Split channels in Figure 5D to show GIP-1 and DNA staining alone. Added colored labels for multicolor image.
10. Added a new panel (E) to Figure 5 containing Images of wild-type and chd-1(bs122); dpl-1 embryos stained for centriole marker SAS-4 to show that extra GIP-1 foci are bonified centrosomes. Updated figure legend and text accordingly.
11. Added data from time-lapse recordings *of chd-1(bs122); dpl-1(bs169)* embryos expressing a *spd-2::mCherry* transgene. These recordings show that the extra centrosomes arise in association with existing centrioles, as expected for an overduplication defect. This data is presented in a new figure (S6). Updated text and figure legends accordingly.
12. Added data to text showing that the timing of the early embryonic cell cycles in *chd-1* null embryos is similar to wild-type embryos.
13. Added quantitative immunoblot to Figure 5, showing SAS-6 levels in *chd-1(bs122)*, *dpl-1(bs169)* and *chd-1(bs122); dpl-1(bs169)* worms. Updated figure legend and text accordingly.
14. Added quantitative immunoblot experiment to Figure 5D showing that depletion of CDK-2 does not affect level of SAS-5. Updated text and figure legends accordingly.
15. Strain table has been updated to include multiple new strains that were used to address reviewers’ questions.
16. Added paragraph to discussion to explain how a reduction of SAS-5 upon loss of CHD-1 impacts phenotypes in the various genetic backgrounds.

**Reviewer #1: In the study titled “The Chromatin Remodeling Protein CHD-1 and the EFL-1/DPL-1 Transcription Factor Cooperatively Down Regulate CDK-2 to Control SAS-6 Levels and Centriole Number”, Iyer et al. identified that SAS-6 levels is regulated through CHD-1 and EFL-1/DPL-1 in C.elegans. The title concludes that centrosome duplication is involved, for the authors to directly conclude this it would be useful to demonstrate over duplication of centrioles using live cell imaging versus a cytokinesis defect. Some of the example images included demonstrate cells with clear supernumerary centrosomes (Fig 5D). If the panel was labeled clearly as a 2-cell stage embryo it could demonstrate that the phenotype was due to over-duplication. However, it would strengthen the study and support the main conclusion in the title to demonstrate overduplication through live-cell imaging**

As suggested, we have added live cell imaging to the manuscript. This data is presented in a new figure (Figure S6) and shows that the extra centrosomes arise in association with existing centrioles and not as a result of cytokinesis failure. This behavior is consistent with an overduplication defect. We also show that these extra centrosomes are capable of duplicating, providing further evidence that they represent bona fide centrosomes.

**It was also intriguing that previous studies found that CHD-1 can have an interaction with the centrosome and that this interaction can potentially confer function**. **The authors herein do not find any interactions of CHD-1 with centrosome proteins through IP analysis. It would be useful for the authors to expand upon these ideas more. For instance, is there differences between the C. elegans centrosome protein composition and Xenopus centrosome that could account for this? Overall, the paper was well written and with expanded discussion and live-cell imaging studies demonstrating over-duplication, it is acceptable for publication at PLOS Genetics.**

As to why CHD-1 localizes to centrosomes in *Drosophila* but not worms, we really don’t have an answer. So far, centrosome localization has only been reported in flies, and thus this might be specific to *Drosophila*. As to the Hatch et al. 2010 paper that reported an interaction between Plx4 (ZYG-1 homolog) and CHD1 in Xenopus, they performed their interaction screen by overexpressing a mutant version of Plx4 (kinase dead) that they postulated might have a more sustained interaction with binding partners. We pulled down CHD-1 using wild-type animals that express ZYG-1 at very low physiological levels. Thus, if this interaction exists in worms, it might fall below the detection limit of our mass spec approach. Alternatively, the interaction described by Hatch et al. might be an artefact of their approach and since they did not follow up on their initial observation, we have no way of knowing if this interaction is real and if it plays a physiological role in regulating centriole assembly in frogs.

**Are there experiments that the authors could perform to distinguish if CHD-1 has a role at the centrosome separate from its role as a chromatin remodeling protein?**

We can’t think of any additional experiments to address this issue, but we did show that CHD-1 requires its helicase activity to regulate centriole assembly (Figure 2D), suggesting it functions as a chromatin regulator in this capacity. We also did not detect CHD-1 at centrosomes in embryos but did find it localizes to nuclei (Figure 2B). All the available evidence suggests that it regulates centriole assembly by controlling gene expression.

**Specific Comments:**
• **190-192, it is claimed that absence of CHD-1 does not cause death or sterility on an organism level, but does it cause defects in mitotic progression on the cellular level that could be discussed?**

Prompted by the reviewer, we performed time-lapse imaging of wild- type and *chd-1(bs185)* embryos expressing gfp::histone and spd-2::mcherry transgenes and analyzed cell cycle progression. Mutant embryos did not differ from their wild-type counterparts in the timing of the early cell cycles. This new data has been added to the paper beginning on line 231.

**• Lines 270-271, its stated that CHD-1 is a low abundance protein based on a band against GFP. Can endogenous untagged CHD-1 be examined?**

To address this question, we would need to raise an antibody to the endogenous protein and then use the antibody to probe worm extracts comparing the amount of the protein in extracts to a standard curve of purified CHD-1. The production of affinity purified sera would take over three months (if it worked). We would then have to validate the antibody and perform the necessary experiments. Since the exact level of CHD-1 does not impact the main findings of our paper, the expense and time to perform these experiments does not seem justified. If the reviewer prefers, we could simply delete our comments on the abundance of CHD-1

**• Model and immuno-fluorescent resolution were low and it was difficult to visualize images. Labeling for these images can be improved upon (e.g. time stamps, what colors mean in images), and separating channels for clear visualization of centrosome components being perturbed or numbers altered (most notably in Figures 1E, 5D).**

We agree that the images in the PDF version of our manuscript were of poor quality. However, we hope that the reviewers are able to utilize the links embedded within the PDF to access the high-resolution versions of our figures. All figures were provided at 600 dpi which at the top of the acceptable range requested by the journal. As suggested by the reviewer, we have added color keys to all multi-channel images and have added time stamps to time-lapse data sets as requested. We have also separated the channels in Figure 5B as suggested. We did not do this for Figure 1E because of space limitations caused by the addition of schematics showing how centrioles behave in the two worm stains.

**• Lines 347-353 (Figure 3A and 3B), expression of SAS-6 and SPD-2 were analyzed. Are other centrosome proteins affected or is CHD-1-loss specific to just these two?**

Yes, in the original version of the manuscript we measured two other centriole assembly factors. In Figure S4B we demonstrate that SAS-5 levels are actually lower in *chd-1* mutants than in the control. We have no explanation for this result other than to say that regulation by CHD-1 is complex and could involve both positive and negative regulation of centriole assembly. We also measured the levels of ZYG-1 at centrioles (we are unable to detect ZYG-1 on westerns). We found a slight increase in centrosome-associated ZYG-1 levels which might be due to the increase in the level of its centriole receptor SPD-2. Finally, in the revised version we also show that centrosome-associated SAS-4 levels are similar in wild-type and *chd-1(bs122)* embryos.

**• Figure 1 E(F), figure description was mislabeled, description of Figure 1F should be for figure 1E.**

Thank you for pointing this out. We have labeled the figure correctly.

**• Figure 5D needs to be better labeled and adding an image of wild type negative control as comparison. Figure legends for Figure 5D was mislabeled as 5E. Different labeling of figure 5D could improve ease of reading. For example: dpl-1 can be changed to dpl-1(bs169)).**

As suggested, we have added control embryos for the immunostaining experiments (new Figure 5E and 5F . We have also added additional labels to to make the data easier to read. As mentioned above, the revised figure also shows single GIP-1 and DNA channels. The figure legend has been corrected**.**

**• Model 6E was difficult to read, consider revising.**

We are not sure if this comment is referring to the resolution of the figure. If it is, downloading the high-resolution version should solve this issue. However, we have added some additional details to our model and hope this also improves the presentation. If not, it would help us if the reviewer could be more specific about what should be revised.

Reviewer #2: Central to cell replication is the duplication of microtubule-based structure called centrioles that lie at the heart of the spindle poles. Although enigmatic, the process of centriole duplication has been genetically dissected over the last two decades, including by work from the O’Connell lab. Here, Iyer et al. investigate the regulation of the expression of the building blocks of the centrioles, uncovering a role for the chromatin remodeling protein CHD-1, the transcription factor EFL-1/DPL-1, and the kinase CDK-2. Mutations in chd-1 suppress lethality associated with the loss of the kinase zyg-1/Plk4 and restore some “normal” centriole duplication. CHD-1 depletion also leads to increased protein levels, by not transcription, of SPD-2 and SAS-6 and acts in parallel to EFL-1/DPL-1 to downregulate the transcription of CDK-2. Loss of CDK-2 leads to the downregulation of SAS-6 protein levels. Thus, the authors propose that CHD-1 and EFL-1/DPL-1 function in parallel to regulate the expression of CDK-2, which in turn promotes SAS_6 expression to directly regulate centriole duplication. The experiments appear well executed and controlled and the topic area provides a conceptual advance. I think the paper would be suitable for publication but recommend a few changes:

**- The authors clearly demonstrate that SAS-6 and SPD-2 levels are upregulated in the various depletion cases (cdk-2, chd-1). However, the connection between the upregulation of these protein levels and the ensuing phenotypes is tenuous. Can the author test whether partial depletion of spd-2 or sas-6 (either the spd-2 ts mutant or RNAi) suppresses the cdk-2 or chd-1 phenotypes**.

We spent months attempting what would appear to be a simple experiment. We tried to control the level of SAS-6 depletion by altering the amount of IPTG used to induce double stranded RNA and by mixing SAS-6 RNAi bacteria with control bacteria in different ratios. After multiple attempts neither proved to work in a reproducible manner. Under all conditions, some animals exhibited extremely high embryonic lethality while others exhibited no lethality. This made interpretation of the results impossible. What we really needed was a temperature-sensitive *sas-6* mutant that we could fine tune by growing at intermediate temperatures. Unfortunately, no such mutant exists. However, we attempted a similar experiment using *the zyg-1(it25ts)* mutation to reverse the embryonic lethality of a *dpl-1(bs169)* mutant. The idea here is that if the overexpression of SAS-6 was responsible for lethality (because of an overactive centriole assembly pathway) we might be able to rescue this defect by turning down the activity of the pathway by partially inhibiting ZYG-1. We found that on average *dpl-1(bs169)* mutants exhibit 37% viability at 20°C while *zyg-1(it25) dpl-1(bs169)* double mutants exhibit 49% viability at this same temperature. While encouraging, this difference did not prove to be statistically significant. However, this difference did not take into account that the *zyg-1(it25)* mutation carries a fitness cost of 10% lethality. Thus, in the absence of a genetic interaction between *zyg-1* and *dpl-1*, one would expect that adding *zyg-1* to the *dpl-1* mutant would decrease viability to 33% (37 x 0.9) but instead it increased the average viability of the *dpl-1* mutant. We have not added this data to the paper as we are unsure how compelling it would be. We think the best course of action is to simply carefully word the manuscript to convey the idea that we can’t solely attribute the embryonic lethality in the *dpl-1* and *chd-1; dpl-1* mutant strains to SAS-6 overexpression.

**- Similarly, the connection between the centriole duplication-based phenotypes and cdk-2 could be strengthened. Does overexpression of cdk-2 on its own produce a centriole overduplication phenotype? Are the effects of cdk-2 specific? For example, loss of CDK-2 following auxin treatment might just lead to cell cycle arrest that would in turn decrease SAS-6 levels. Are other proteins (maybe SAS-5) unaffected in this depletion case? Do the authors see the same enhancement of the chd-1/zyg-1 or dpl-1/zyg-1 phenotype following loss of CDK-1 for example?**

One of our long-term goals is to engineer a system to overexpress factors like SAS-6 and CDK-2 in the early embryo. As there is a good chance that overexpression of these factors would be lethal, we would need an inducible system. We would also need to find a promoter/3’ utr pair that is substantially stronger than the endogenous cdk-2 promoter/3’utr. Since it would take a significant amount of time to engineer these constructs with no guarantee of success, we decided to prioritize other experiments. However, as mentioned in the manuscript, inhibition of CKI-2, an inhibitor of CDK-2 has been reported to result in supernumerary centrosomes (Kim DY, Roy R 2006) providing independent support for the notion that elevated CDK-2 activity can drive centriole over duplication in *C. elegans*.

The reviewer was also concerned that the effect of depleting CDK-2 on SAS-6 levels might not be specific. As suggested, we analyzed the effect of CDK-2 depletion on SAS-5 levels. Unlike SAS-6, SAS-5 was unaffected. This argues that the loss of CDK-2 does not indirectly affect the levels of centriole assembly factors by disrupting the cell cycle. We have included this new data in Figure 6D.

As for exploring a potential effect of CDK-1 depletion on viability of the *chd-1; zyg-1* or *dpl-1: zyg-1* double mutants, we don’t feel the results of such an experiment would influence our interpretation of CDK-2 function one way or the other. For instance, if depletion of CDK-1 did result in loss of viability of one or the other of the double mutants, this might suggest a role for CDK-1 in centriole assembly. However, this would not mean that the effect of CDK-2 is nonspecific. We agree that by itself, our finding that depletion of CDK-2 reverses suppression of *zyg-1(it25)* by *dpl-1* or *chd-1* mutations would not mean much. However, taken together with the rest of our data, we feel that we present a compelling case that CDK-2 regulates centriole assembly via controlling SAS-6 levels. Just to summarize, we found that loss of either DPL-1 or CHD-1 suppress a *zyg-1* mutant, that *cdk-2* transcripts and SAS-6 protein levels are elevated in both mutants, that partial depletion of CDK-2 lowers suppression of *zyg-1* by both *dpl-1* and *chd-1* mutations, and that depletion of CDK-2 results in downregulation of SAS-6 (but not SAS-5) levels.

**- Figure 1E: needs a better description of duplication process to make these images understandable to non-C. elegans people. The images need time stamps and indication that the centrosomes and not centrioles are shown and the relation of the results here to centriole duplication defects.**

Thank you for this nice suggestion. We have added schematics to Figure 1E to explain the duplication process in each strain. We have also added time stamps and a color key to emphasize that we are monitoring centrosomes via SPD-2::mCherry fluorescence.

**- Although likely chronological, I find the points of Figure 3D and E detracting from the main thread of the story. Figure 3F supports the main conclusion and Figure 3D and E show that endogenous flanking sequence is important. I would switch the order of the presentation of this information and perhaps move 3D and E to the supplement.**

As suggested by the reviewer we have moved 3D and 3E to supplemental figure 5. We agree with the reviewer’s opinion that the homeotic SPD-2 control mechanism and the role of the flanking region detracts a bit from the main story. However, changing the order of presentation creates its own problems. We therefore decided to leave the narrative as is.
 **- Line 479: suggesting that overexpression of SAS-6 is toxic—this is a leap based on the information presented here as I am sure many transcripts are affected following the loss o**f **both CHD-1 and DPL-1**

We only meant to offer this scenario as one possible explanation. We have tried to re-word the manuscript as carefully as possible to avoid the impression that we are concluding such.


**- Can you quantify the effects seen in Figure 5D: in the one example of dpl-1 single mutant, supernumerary centrosomes appear to coalesce at the poles while the chd-1;dpl-1 double appear multipolar. Is this the case?**

We apologize for any confusion, but we did not observe any instance of centrosome clustering at the spindle poles in either the *dpl-1(bs169)* single mutant or the *chd-1(bs122); dpl-1(bs169)* double mutant. All images are maximum intensity projections and so while some centrosomes might appear to be clustered in these two-dimensional images, they are in fact well separated in the Z-axis. We amended the figure legend to make it clear to the reader that the images are projections. Also, we should point out that we made a mistake in labeling the original figure. The embryo in Figure 5D that was previously labeled as *dpl-1* is actually a double mutant and the one below it that was labeled as a double mutant is actually a *dpl-1* mutant. We apologize for this embarrassing error and have fixed it in the new version of Figure 5. Please note that in our amended manuscript, what was previously Figure 5D is now Figure 5E.

Minor points:

**- There are several instances where data is referenced but not shown:
1.Where is the data for lines 205-210?**

At first, we did not feel it was necessary to photo-document these results because they are somewhat redundant with that data shown in panels D and E of the original version of Figure 1. However, given the reviewer’s concern, we have decided to add select frames from time-lapse DIC movies to Figure S1.

**2.Where are the IP data, i.e. the gel? How can we assess the legitimacy of the results of this experiment without being able to assess if the IP worked.**

We have added an image of an immunoblot of our pull down to Figure S2 to allay any concerns the reviewer might have. Importantly however we were able to validate our pull down by showing that CHD-1 peptides were only detected in our CHD-1::GFP pulldown and were not detected in the control GFP pulldown. This data has been added to Figure S2B.

**3. Where is the data to support the diagnosis of successful cytokinesis (line 493-496)**

The hallmarks of cytokinesis failure in *C. elegans* embryos are the presence of binucleate cells and an even number of excess centrosomes (for example: http://www.wormbook.org/chapters/www_celldivision/celldivision.html). In examining *dpl-1* and *chd-1;dpl-1* mutants we did not detect a single binucleate cell. We have added a line to Table 1 to emphasize that of the 46 *dpl-1* embryos and 48 *chd-1;dpl-1* embryos (all at the 2-6 cell-stages), that we examined, we observed 0% with binucleate cells. We did not include one-cell zygotes in this analysis because they naturally have two nuclei (the male and female pronuclei). However, one cell embryos were also informative in this regard. If cytokinesis failure were occurring in these mutants, we would expect to observe some one-cell stage embryos with four centrosomes due to a failed first division. However, as indicated in Table 1, all one cell embryos had two centrosomes. Lastly we should point out that our failure to see defects at the one cell stage is consistent with a maternal overduplication phenotype such as described for PP1 mutants (Peel et al 2017 DOI: [10.1371/journal.pgen.1006543](https://doi.org/10.1371/journal.pgen.1006543)) where bipolar spindles are observed at the one-cell stage and multipolar spindles only at later cell stages. Finally, we have added live imaging experiments (new figure S6) to show that extra centrosome arise in association with pre-exiting centrioles (as expected for an overduplication defect) and not through cytokinesis failure.

**- Figures are low resolution. The images are particularly hard to interpret at this resolution. This may just be the reviewer copy, but I would make sure that the final veriosn of the figures are at higher resolution. I would also encourage the authors to include larger and higher resolution images of their photographic data throughout. There is a lot of wasted white space in the figures that could be used for making images larger without having to make the overall figures bigger.**

Reviewer 1 also made a similar comment. As mentioned above, we agree that the images in the PDF were of low resolution and hope that that the reviewers are able to access the links embedded in the PDF to download high-resolution copies of each figure. All figures were provided at 600 dpi which it as the high end of the scale specified by the journal. As suggested by the reviewer, we have made images larger where possible.

**- Figure S3A - SAS-4 also seems upregulated. Is this the case?**

We had wondered this ourselves and therefore quantified centrosome-associated SAS-4 levels in both wild-type and *chd-1(bs122)*. However, we did not find a significant difference between the two strains. For this reason, we did not include the quantitation in the original submission but it’s now clear that some readers might be curious about this, so we added the SAS-4 quantitation to Figure S5

**- Line 90: in a mice**

Fixed.

**- Figure 1D: This should be represented as % events rather than number of events**

We have modified this panel accordingly.

**- Line 357 needs a comma**

Fixed.

**- Line 488: please define GIP-1 ortholog, i.e. g-TuRC component GCP3**

Done.

Reviewer #3: This is a very interesting study that explores the regulation of centriole number maintenance by transcriptional control pathways . In this manuscript Iyer and colleagues identify two pathways working in parallel and converging on the kinase CDK-2, which in turn regulates SAS-6 protein levels to prevent centriole overduplication in C. elegans. The authors identify CHD-1 as one of the regulatory pathways by a suppression phenotype of the zyg-1(it25) mutation and devote substantial effort to analyze its role in the regulation of centriole core components. Further, they demonstrate that the E2F-DPL-1 complex acts in parallel to CHD-1 in the same process. This study is in line with previous works demonstrating a role of CDK-2 in centriole assembly in vertebrates, but it adds a mechanistic view to the regulation. I think the paper is of high interest. Nevertheless, the authors should address the following concerns before the manuscript can be considered suitable for publication in PLOS Genetics.

**Major comments:
1. On page 16, the authors engineer a mutant to test the requirement of the ATPase activity of the CHD-1 helicase and come to the conclusion that it is required for suppression of the zyg-1(it25) lethality. On page 29 authors mention that in other systems CHD1 can have functions which are not associated with the chromatin regulation. I wonder if the authors tried to disrupt the DNA-binding domain in CHD-1 and to test whether this would have a comparable effect as the mutation in the ATPase domain. Such an experiment could help to discriminate whether the regulation of centriole biogenesis is indeed through chromatin.**

We did in fact make a strain with a deletion of the DNA-binding domain and surprisingly this mutation did not have any effect on *zyg-1(it25)* lethality. However, the role of the DNA-binding domain in CHD-1 function is a bit unsettled. One in vitro study suggests that it is important for chromatin remodeling while in vivo work suggest it may not be essential for CHD-1 function. Given our results and the ambiguous role of the DNA-binding domain in chromatin remodeling, we decided that adding the data to the paper would not help resolve the question of a non-chromatin remodeling role for CHD-1 in the regulation of centriole duplication. In contrast, the role of the helicase domain in chromatin remodeling is very well established, and thus the *chd-1(bs123)* mutation that is expected to inactivate the helicase activity is really the most suitable mutant that can be employed to address this question.

**2. On pages 16-17, the authors find that in chd-1(bs122) mutants protein levels of SAS-6 and SPD-2 are upregulated. The authors then explore in detail the regulation of SPD-2 levels at the centrosome using different constructs and come to the conclusion that SPD-2 is upregulated through its promoter and 3’UTR. These experiments are done in the presence of the endogenous protein, which complicates the analysis. I wonder why the authors did not use an available endogenously CRISPR-tagged protein or immunostainings to measure endogenous SPD-2 levels at the centrosome. Alternatively one could down-regulate endogenous SPD-2 by RNAi given that the SPD-2:mCherry transgene is RNAi-resistant.**

The simple answer is that we had the transgenic strains on hand and live imaging is much more efficient and far less labor intensive than immunofluorescence. Further, using fluorescently tagged transgenes to measure the levels of proteins at the centrosomes is a long-standing acceptable method. Also, had we just used immunostaining, we would not have been able to deduce that CHD-1 functions through the *spd-2* flanking regions. Nonetheless, we feel that our data clearly shows that loss of CHD-1 results in an elevation of SPD-2 levels at the centrosome but only when using the endogenous *spd-2* promoter and 3’ utr to drive expression.

**3. It is puzzling that the elevated SAS-6 protein level in chd-1(bs122) does not translate into higher levels of SAS-6 at the centrioles. This could be due to the fact that only a finite number of SAS-6 molecules can be incorporated in a given centriole or alternatively the decreased SAS-5 protein level could be a limiting factor. The authors should at least discuss how the decreased SAS-5 levels fit into the model of elevated SAS-6 levels in chd-1(bs122) single mutants**.

Please see answer to next point.

**4. While the findings of elevated SAS-6 overall levels in the chd-1(bs122) mutants is an important result, the authors should test for SAS-5 and SAS-6 levels at the centrioles in the sensitized chd-1(bs122) zyg-1(it25) double mutant background, where an increase might be more evident.**

Thank you for this nice suggestion. We measured SPOT::SAS-6 levels in a sensitized *zyg-1(it25)* background. Interestingly, we found that in this background, the *chd-1(bs122)* mutation did indeed result in a statistically significant increase in the levels of centrosome-associated SAS-6. Thus, we can now say that loss of CHD-1 results in increased total levels of SAS-6, increased levels of centrosome-associated SAS-6, and suppression of the *zyg-1(it25)* mutant. The new data is presented in Figure 3 and some discussion of why we might not have observed an increase of centrosome-associated SAS-6 in the *zyg-1* wild-type background is presented in the paragraph beginning on line 384.

**5. The authors demonstrate that chd-1(bs122) and dpl-1(RNAi) in an otherwise wild type background (non-sensitized) have a synergistic effect on embryonic viability and that chd-1(bs122) enhances the lethality of dpl-1(bs169). This strongly indicates that in the double mutants SAS-6 protein levels are even more elevated and reach a toxic level. If this is the underlying cause of centriole overduplication, authors should demonstrate elevated SAS-6 protein levels in this combined genetic background.**

As suggested by the reviewer we performed quantitative immunoblots to measure the relative levels of SAS-6 in *chd-1(bs122)*, *dpl-1(bs169)*, and *chd-1(bs122); dpl-1(bs169)* worms. Consistent with our expectations, we found that SAS-6 was elevated 1.8-fold in the double mutant relative to the *chd-1* single mutant. We also found a smaller 1.3 fold increase in SAS-6 levels in the double mutant relative to the *dpl-1* single mutant, although this difference did not achieve statistical significance. Thus, the double mutant expresses more SAS-6 than the *chd-1*single mutant, and possibly somewhat more that the *dpl-1* mutant. This data has been added to Figure 5D.

**6. The authors claim to see supernumerary centrosomes in the chd-1;dpl-1 double mutants. While this is a very important and exciting finding, some more evidence is needed. Extra gamma tubulin foci can be a consequence of different processes like fragmentation of the PCM, fragmentation of centrioles, premature disengagement of both or one centriole pair or failed cytokinesis. The authors argue that the extra gamma-tubulin foci are only detectable from the second division on and that the absence of binucleated cells indicates a successful cytokinesis. It is difficult to draw a conclusion using only gamma tubulin as a marker and fixed immunostaining samples. Especially the image of the dpl-1 single mutant (Fig. 5D top right) shows a small and big gamma tubulin focus, which resembles a prematurely disengaged centriole pair. To conclude that the extra foci are for sure a consequence of centriole overduplication, authors need to provide some more evidence. They could use additional centriole markers and/or perform live cell imaging to precisely determine when and where these extra foci/centrioles appear.**

As suggested we have reanalyzed the *chd-1;dpl-1* double mutants by immunostaining for the centriole marker SAS-4. As shown in Figure 5F, SAS-4 can be found at all poles of multipolar spindles, consistent the presence of centrioles. Further we have measured the intensity of SAS-4 at each pole of multipolar spindles and at both poles of control spindles. If centrioles were fragmenting, we would expect that some poles of a multipolar spindle would have significantly weaker SAS-4 signal than the other poles. However as presented on line 542, we found that multipolar spindles did not vary in SAS-4 intensity anymore than wild-type bipolar spindles, arguing against centriole fragmentation. Regarding the image mentioned by the reviewer (original Figure 5D) which appears to show a cell with one large pole and one small. We should point out that these images are maximum intensity projections, and this appears to be an instance where one of the centrosomes was much closer to the cover slip while the other was buried deep in the embryo. In these cases, the deeper centrosome will always be dimmer. We have now specified in the figure legends that the images are projections. Finally, we have performed live cell imaging experiments as suggested and find that the extra centrosomes arise in association with pre-existing centrioles as expected for an overduplication defect. In these experiments we did not observe cytokinesis failure.

**7. In the discussion no explanation is offered for some of the rather surprising and puzzling results. For instance, how do the authors reconcile the decreased SAS-5 levels in chd-1(bs122) with the model of centriole overduplication and how do the authors explain the discrepancies in the RNAseq and the qRT-PCR data?**

We have added a paragraph to the discussion to explain how the various phenotypes observed in the mutants might be explained considering the effect of *chd-1(bs122)* mutation on SAS-5 levels. As for what appears to be a discrepancy between RNAseq and qRT-PCR, there is a simple explanation. The fold change in *cdk-2* mRNA levels as measured by qRT-PCR was 1.4 whereas the threshold for determining fold change in the RNA-Seq was 2. There is no discrepancy at all, just a matter of the magnitude of the change. Also, qRT-PCR is considered the gold standard in measuring transcript levels, as it is far more accurate at detecting subtle changes in mRNA levels than RNA-Seq.

**Minor issues:
1. Figure 1 the labels of D, E and F need to be adjusted according to the figure legends.**

Done.

**Figure 1E would be better represented in percentages, as a stacked column. In Figure 1F it would be helpful if the authors add the time stamps to the stills.**

We have made both suggested changes.

**2. In Figure 2B the insets are hardly visible, they can be increased in size.**

Done.

**3. Line 324/Figure S2 Authors state that CHD-1(D538N)::sfGFP localization is not altered. In the images it seems that the mutant version localizes much stronger to the nuclei of the oocytes than the wild type protein. Authors should comment on this in the text.**

We did not observe any difference in intensity between oocyte nuclei in wild-type and mutant worms. The images in this figure (now Figure S3) are single optical slices (projections are not feasible due to the complexity of the germ line) The image we chose for the wild-type worm nicely displays the distal germ line nuclei but unfortunately this optical plane does not contain oocyte nuclei. However, the expression of the wild-type fusion protein in oocyte nuclei can be seen in the insets of Figure 2B. To address this issue, we now point this out in the figure legend.

**4. In Table 1 an indication of the present (%) of for a given phenotype would be helpful.**

Done.

**5. For figure S3 it should be specified in the methods which SAS-5 antibody was used and which isoform was quantified to measure SAS-5 levels.**

Thank you for pointing out this omission. We have added a description of the SAS-5 antibody to the Materials and Methods section. We also specify in the legend to this figure (now figure S4) that the quantitation pertains to the SAS-5a isoform.

**6. In Figure 5D image of the embryos can be increased for better visibility.**

We did increase the size of the images as much as possible, but in response to another reviewer’s request we split the channels so that we could show the GIP-1 and DNA staining separately. This increased the number of images and restricted our ability to futher increase the size of individual images.

**7. For completion the authors could include an arrow for the non-CDK-2 input of CHD-1 in the model.**

We have revised this model and have included the requested arrow.
